# Epigenetic Regulation of Neural Stem Cells in Developmental and Adult Stages

**DOI:** 10.3390/epigenomes8020022

**Published:** 2024-06-04

**Authors:** Shu Kunoh, Hideyuki Nakashima, Kinichi Nakashima

**Affiliations:** Department of Stem Cell Biology and Medicine, Graduate School of Medical Sciences, Kyushu University, Fukuoka 812-8582, Japan; kunoh.shu.488@s.kyushu-u.ac.jp

**Keywords:** neural stem cells, epigenetics, brain, development, neurogenesis, astrogenesis, chromatin

## Abstract

The development of the nervous system is regulated by numerous intracellular molecules and cellular signals that interact temporally and spatially with the extracellular microenvironment. The three major cell types in the brain, i.e., neurons and two types of glial cells (astrocytes and oligodendrocytes), are generated from common multipotent neural stem cells (NSCs) throughout life. However, NSCs do not have this multipotentiality from the beginning. During cortical development, NSCs sequentially obtain abilities to differentiate into neurons and glial cells in response to combinations of spatiotemporally modulated cell-intrinsic epigenetic alterations and extrinsic factors. After the completion of brain development, a limited population of NSCs remains in the adult brain and continues to produce neurons (adult neurogenesis), thus contributing to learning and memory. Many biological aspects of brain development and adult neurogenesis are regulated by epigenetic changes via behavioral control of NSCs. Epigenetic dysregulation has also been implicated in the pathogenesis of various brain diseases. Here, we present recent advances in the epigenetic regulation of NSC behavior and its dysregulation in brain disorders.

## 1. Introduction

**Figure 1:**
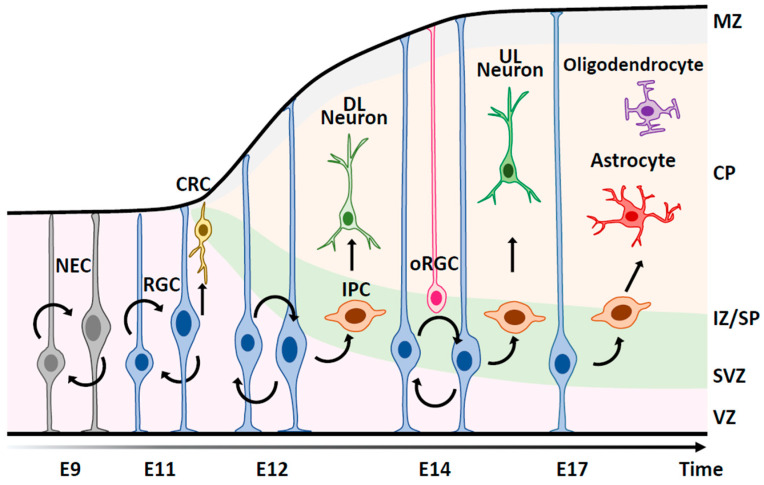
A schematic illustration of spatiotemporal development of the cerebral cortex in mice. Neuroepithelial cells (NECs) undergo symmetric cell division to produce an initial pool of cortical progenitors that later transform into radial glia cells (RGCs), which act as primary neural stem cells (NSCs). RGCs first produce Cajal–Retzius cells (CRCs), which form the preplate. RGCs next produce deep layer (DL) neurons (layer VI/V), and subsequently upper layer (UL) neurons (layer IV/III/II) mostly through intermediate progenitor cells (IPCs). In later stages, RGCs acquire the ability to differentiate into glial cells and give rise to astrocytes and oligodendrocytes. MZ, the marginal zone. CP, the cortical plate. IZ/SP, the intermediate zone/subplate. SVZ, the subventricular zone. VZ, the ventricular zone. oRGC, the outer radial glia cell.

**Figure 2:**
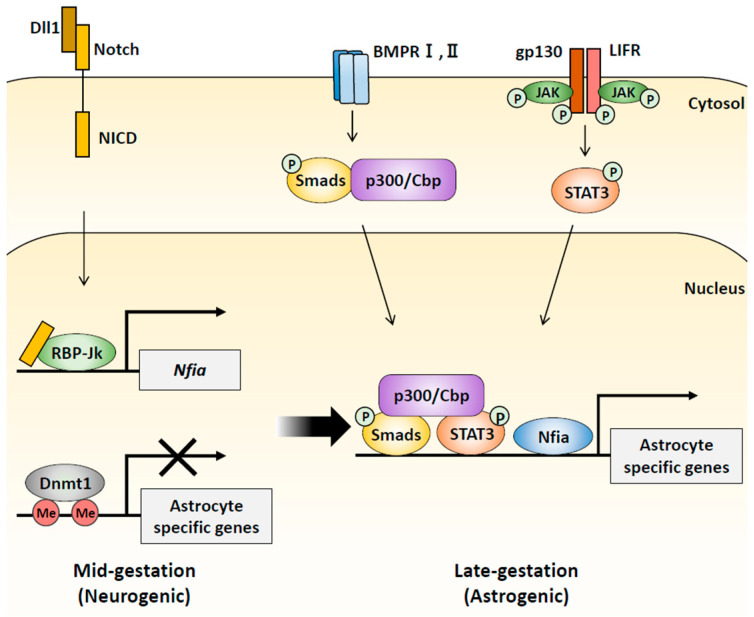
Schematic illustration of stage-dependent epigenetic alterations in NSCs. In mid-gestation, the neuronal-committed precursors (neuroblasts) and newly generated immature neurons expressing Notch ligands such as DLL1 activate Notch signaling in adjacent NSCs to produce cleaved Notch (NICD). The released NICD then binds to RBP-Jk and activates *Nfia* expression. Nfia dissociates Dnmt1 from the astrocyte-specific promoters, resulting in demethylation in the region. In late gestation, astrocyte-specific gene promoters are demethylated, and thus both the JAK-STAT3 pathway and BMP signaling cooperate to synergistically induce astrocyte-specific genes through STAT3 and Smads forming a complex via P300/CBP.

The brain is a complex organ with a highly specialized structure that, in humans, contains about 100 billion neurons and one trillion glial cells [1]. Neural stem cells (NSCs) are defined as cells that can self-renew and differentiate into three cell types: neurons, astrocytes, and oligodendrocytes [2]. This multiple differentiation potential of NSCs is gradually obtained during development through highly organized processes (Figure 1). In the early stages of neocortical development, NSCs divide symmetrically to expand their pool and then switch to asymmetric divisions to give rise to neurons at mid-stages [3]. In the late stages of neocortical development, NSCs acquire a gliogenic potential to generate astrocytes and oligodendrocytes [4,5]. These changes in the differentiation potential of NSCs during developmental progression are strictly regulated by the coordination of extracellular factors and epigenetic mechanisms (Figure 2). They are critical for the generation of a balanced number of each neural cell type for proper neuronal circuit formation [6].

**Figure 3:**
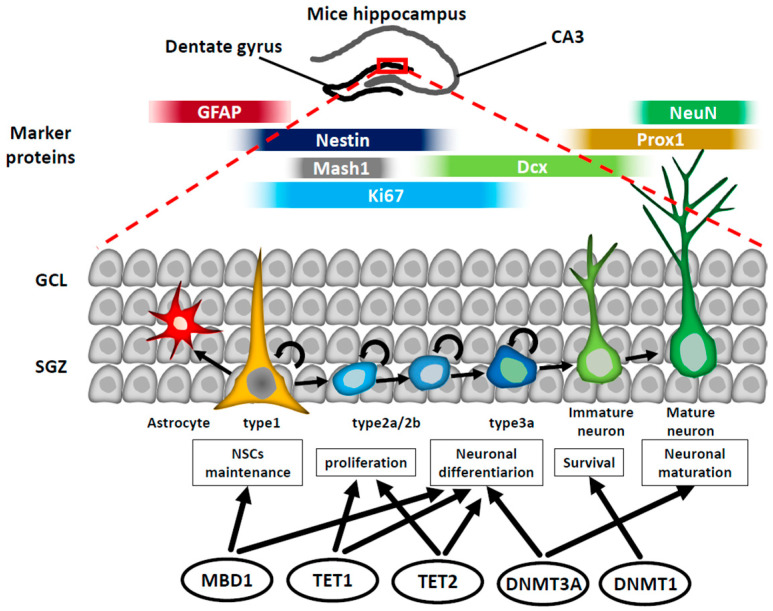
Process of adult neurogenesis in mouse hippocampus. Under physiological conditions, NPCs produced from adult neural stem cells (aNSCs) in the subgranular zone (SGZ) of the hippocampal dentate gyrus sequentially turn into type 2a/2b cells and type 3 cells. Then, these cells differentiate into immature neurons, which mature into neurons and are incorporated into existing neuronal circuits. MBD1; Methyl CpG Binding Domain Protein 1, TET1; Ten-eleven translocation 1, TET2; Ten-eleven translocation 2, DNMT1; DNA methyltransferase 1, DNMT3A; DNA methyltransferase 3 alpha.

**Figure 4:**
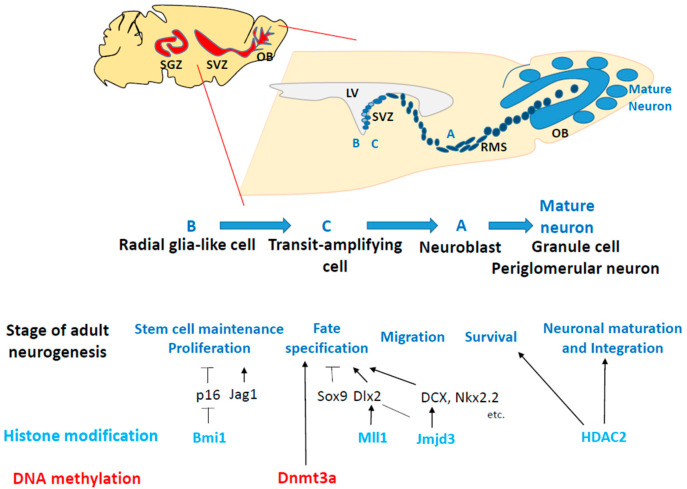
A diagram illustrating the process of adult neurogenesis in the subventricular zone (SVZ) and olfactory bulb (OB), along with the factors influencing epigenetic regulation. Neurogenesis in the adult SVZ consists of five stages: (1) maintenance and proliferation of stem cells, (2) determination of cell fate, (3) migration, (4) survival, and (5) maturation and integration of neurons. In brief, activated radial glia-like cells (type B) generate NPCs (type C), which undergo frequent proliferation to give rise to neuroblasts (type A). The neuroblasts then travel along the rostral migratory stream and undergo maturation into immature neurons in the olfactory bulb. Subsequently, synaptic integration and maturation take place in both granule cells and periglomerular neurons within the olfactory bulb. The diagram also illustrates key epigenetic factors involved in regulating each phase of adult neurogenesis in the SVZ. LV, lateral ventricle; OB, olfactory bulb; RMS, rostral migratory stream. → indicates promotion, whereas ⊣ indicates inhibition.

After brain development is completed, NSCs persist in the adult brain throughout an individual’s entire lifetime in two well-established regions: the ventricular–subventricular zone (V-SVZ) lining the lateral ventricle and the subgranular zone (SGZ) of the dentate gyrus (DG) in the hippocampus (Figure 3 and Figure 4; see later sections for details) [7,8,9]. Hippocampal NSCs generate excitatory granule cells, which are integrated into the hippocampal circuitry and contribute to cognitive functions, and are involved in several neurological disorders, including age-related cognitive decline, major depressive disorders, and medial–temporal lobe epilepsy [10,11]. Thus, elucidating the mechanisms underlying the regulation of neurogenesis in NSCs is important for developing therapeutic strategies to overcome diseases caused by impaired adult neurogenesis.

The term “epigenetics” was coined by Conrad Waddington in 1942 to describe the complex, dynamic interactions between the developmental environment and the genome that lead to the generation of phenotypes [12]. Various types of epigenetic regulations, such as DNA methylation, histone modifications, RNA methylation, and noncoding RNAs, have long been acknowledged to be critically important for the maintenance and differentiation of stem cells [13,14]. In addition, multiple research studies have shown that different epigenetic elements interact with each other and play an important part in regulating NSC function in cooperation with extracellular cues. In this review, we summarize the recently accumulated evidence revealing mechanisms of epigenetic regulation in the behaviors of embryonic and adult NSCs.

## 2. Outline of Neural Cell Production during Cortical Development

The cells that make up the mammalian brain are generated from NSCs in the ventricular zone (VZ) of the embryonic brain [15]. NSCs have the ability to self-renew and differentiate into the three major cell types of the nervous system (i.e., neurons, astrocytes, and oligodendrocytes) (Figure 1). NSCs do not have these abilities from the beginning but acquire them in a developmental stage-dependent manner [6]. During mouse brain development, NSCs preferentially produce neurons in the cortex from embryonic day 11 (E11) to E18 [16]. In the process of generating these cortical neurons, Cajal–Retzius neurons that form the preplate are first generated. The preplate is then separated into the marginal layer, which later becomes cortical layer I, and the subplate layer. Next, upper layer (UL) neurons (layer II/III/IV) and deep layer (DL) neurons (layer V/VI) are produced in an inside-out order between layer I and the subplate layer as development progresses. As developmental stages progress, NSCs gradually acquire the ability to generate astrocytes. Most mouse oligodendrocytes are generated after birth in the cerebral cortex. These sequential steps first build the neuronal network and then integrate the glial cells that support the function of the neuronal network (Figure 1).

### 2.1. Histone Modification during Cortical Development

The remodeling of nuclear chromatin structure by histone modifications plays an essential role in regulating gene expression. Chromatin consists of multiple nucleosomes composed of DNA and histones. The nucleosome is a fundamental unit of chromatin, in which 145–147 base pairs of DNA wrap around the histone octamer, composed of one H3-H4 tetramer and two H2A-H2B dimers [17]. Histone tails are highly basic due to numerous lysine and arginine residues and play an important role in nucleosome structural stability and chromatin compaction [18]. The N-terminal amino acid residues of histone proteins undergo multiple post-transcriptional modifications, including methylation, acetylation, ubiquitination, SUMOylation, and phosphorylation [19,20,21,22,23]. Changes in chromatin structure due to histone modifications affect gene expression through allowing transcription factors to reach binding factors. The effect of histone methylation on gene expression, for example, depends on the location of the histone tail and the number of methylations on the lysine residues. Histone H3 methylation at lysine 9 (K9) and K27 is associated with gene silencing, whereas the methylation at K4, K36, and K79 induces gene activation [24]. These various histone modifications around both neurogenic and gliogenic gene promoter loci in NSCs allow the consecutive generation of each cell type at the proper stage of development.

In early gestation, ectopic expression of stabilized β-catenin (a constitutively active mutant β-catenin that activates the canonical wingless-type [Wnt] pathway) promotes the proliferation of NSCs, resulting in an enlarged embryonic cortex. In contrast, in mid-gestation, activation of the canonical Wnt pathway signal induces NSC differentiation into neurons through the generation of transiently amplifying neuronal precursor cells (NPCs) by upregulation of a proneural basic helix–loop–helix (bHLH) transcription factor, neurogenin1 (Neurog1) [25]. Altered methylation patterns at histone tails contribute to this stage-dependent control of various responses to extracellular Wnt signaling. The polycomb group (PcG) complex catalyzes H3 K27 trimethylation (H3K27me3) and alters the local heterochromatin arrangement, resulting in transient transcriptional repression [26,27]. PcG is composed of two complexes, polycomb repressive complex 1 (PRC1) and PRC2. PRC2 is responsible for initiating gene silencing by catalyzing the formation of H3K27me3, which serves as a marker to recruit PRC1 for gene silencing. In early gestation, Ezh2, the histone methyltransferase of PRC2, is highly expressed in Neural Stem/Precursor Cells (NS/PCs) and inhibits differentiation into neurons by blocking Wnt signaling-mediated activation of neuronal genes. Toward mid-gestation (neurogenic phase), the expression level of Ezh2 gradually decreases, thereby promoting Wnt signaling-mediated neuronal differentiation of NS/PCs. The conclusion that reduced Ezh2 expression contributes to the acquisition of neuronal differentiation potential in NSCs is supported by the fact that NSC-specific Ezh2 conditional knockout (cKO) mice overproduce early-born neurons from NSCs at the expense of self-renewal at early embryonic stages [28,29]. Moreover, recent single-cell (sc) RNA-seq studies suggested that NSCs abundantly express the chromatin organizer factors Ezh2, Suz12, and Eed, a regulatory subunit of PRC2 that delays cell cycle termination [30]. Neuronal differentiation occurs before glial differentiation of NSCs, and once glial differentiation begins, NSCs gradually terminate neuronal cell production during development. The PcG complex is also involved in the switching of the neurogenic to gliogenic phase. Ezh2 represses Neurog1 expression by catalyzing H3K27 trimethylation in the Neurog1 promoter region [31]. This ultimately leads to the limited ability of NSCs to respond to Wnt signaling, contributing to a switch in the differentiation capacity of NSCs from neurogenic to gliogenic.

Methylation at lysine 9 of histone H3 (H3K9) is generally associated with transcriptional repression of neighboring genes and is also essential for early neural differentiation. ERG-associated protein with SET domain (Eset; also called Setdb1), a histone H3K9-specific methyltransferase, represses gene expression by interacting with the co-repressor KAP1 (Trim28) [32]. In the early stages of cortical development, Stedb1 is highly expressed in proliferating NS/PCs, but not in neuronal layers. However, Setdb1 is downregulated over time and hardly detectable at E17 when the transition from neurogenesis to astrogenesis occurs in the dorsal cortex. NSC-specific or forebrain-specific loss of Setdb1 impairs neurogenesis and promotes astrocyte production [33], suggesting that H3K9 methylation is critical for the proper timing of the switch from neurogenesis-to-astrogenesis of NSCs.

In contrast to methylation of H3K27 and H3K9, methylation of H3K4 is generally associated with transcriptional activation of neighboring genes. In brain development, Lysine-specific H3K4 demethylase 1 (LSD1) is highly expressed in the VZ and SVZ [34]. Inhibition or loss of LSD1 expression in NSCs reduces proliferation through upregulation of cell-cycle inhibitor p21 and downregulation of Atrophin1 (ATN1) [34,35]. In addition, consistent with these studies, LSD1 co-repressor Rcor2 regulates NSC proliferation by repressing sonic hedgehog (Shh) signaling during cortical development [36]. Moreover, recent studies suggested that PHD finger protein 21B (Phf21b) is highly expressed in the neurogenic phase of cortical development and promotes cell cycle termination and neuronal differentiation by recruiting both LSD1 and histone deacetylase Hdac2 to cell cycle-related gene promoters [37]. PR-domain containing 16 (PRDM16) is a transcription factor with conserved PRDI-BF1 and RIZ homology (PR) domain and is known as H3K4 and H3K9 methyltransferase [38,39]. In brain development, Prdm16 is predominantly expressed in the cortical VZ and regulates the epigenetic state of transcriptional enhancers of NSCs to instruct the production of intermediate progenitor cells (IPCs) and neuronal migration [40]. Mechanistically, PRDM16 suppresses the expression of target genes related to neuronal specification, cell cycle regulators, and neuronal migration by limiting chromatin accessibility of the permissive enhancers [41].

Histone acetylation is the most well-defined histone modification and can occur on the lysine residues on the N-terminal tails of H3 and H4. In general, acetylation of histone tails by histone acetyltransferases (HATs) reduces the electrostatic affinity between DNA and histone, resulting in an open-chromatin structure that leads to the transcriptional upregulation of genes. Conversely, histone deacetylation by histone deacetylases (HDACs) induces transcriptional suppression. So far, 18 HDACs have been identified in mammals, and based on domain organization and sequence homology with yeast, HDACs have been classified into four classes (classes I to IV). Both the class I and class II HDACs form inhibitory complexes with LSD1 and repress the cell-cycle inhibitor p21 gene in NSCs [42]. Moreover, treatment of NSCs with a class I and II HDAC inhibitor (valproic acid, VPA) increases the expression of neurogenic transcription factors such as Neurog1, Neurog2, and NeuroD1 by increasing histone acetylation levels around their promoters, which in turn promotes neuronal differentiation [4,43]. Although valproic acid (VPA) is one of the most prescribed antiepileptic drugs in adults, prenatal VPA exposure due to maternal VPA use during pregnancy has been reported to alter the transcriptomic profile of NSCs in both developing and adult brains [44] and increase the risk of neurological diseases such as autism spectrum disorder [45]. These results indicate that precise regulation of histone acetylation status is critical for proper brain development. Histone modification-related genes during cortical development are summarized in Table 1.

### 2.2. DNA Modification during Cortical Development

DNA methylation is an important process in embryogenesis and normal development and one of the most frequently studied epigenetic modifications. DNA methylation occurs predominantly on the cytosine residue within CpG dinucleotides of the mammalian genome. The process of DNA methylation is catalyzed by DNA methyltransferase, mainly Dnmt1, Dnmt3a, and Dnmt3b. Dnmt3 enzymes are de novo methyltransferases [46] and Dnmt1 is mainly involved in the maintenance of DNA methylation in dividing somatic cells [47]. *Dnmt1* or *Dnmt3a* and *Dnmt3b* knockout mice die in mid-gestation, indicating that these genes are essential for proper development.

NSCs sequentially acquire the ability to differentiate into neurons and glial cells during cortical development in response to a combination of spatiotemporally regulated exogenous factors and cell-intrinsic epigenetic changes. The later onset of astrogenesis is attributed to dramatic changes in DNA methylation at astrocyte-specific promoters. Many studies have shown that cytokine signaling pathways contribute to the regulation of astrocyte differentiation from NSCs. The interleukin 6 (IL-6) family of cytokines, including leukemia inhibitory factor (LIF), cardiotrophin-1, and ciliary neurotrophic factor (Cntf), activate the Janus kinase (JAK)/signal transducer and activator of transcription (STAT) pathway through their interaction with a heterodimeric receptor complex of LIF receptor beta and glycoprotein 130 (Gp130), triggering the differentiation of NSCs into astrocytes [48]. Bone morphogenetic proteins (BMPs) also activate the expression of astrocytic genes via the formation of a complex between BMP-downstream transcription factor mothers against decapentaplegic homolog 1 (Smad1) and Stat3 bridged by a transcriptional coactivator, histone acetyltransferase p300/Cbp [49]. Even though the JAK-STAT3 pathway is activated in NSCs in early- to mid-gestation, when NSCs differentiate into neurons only, treatment with IL-6 family cytokines such as LIF does not induce astrocytic differentiation in NSCs [50]. This is due to the DNA methylation of the promoter region containing STAT3-binding sites of astrocytic genes, such as *Gfap* and *S100 calcium-binding protein B* (*S100β*). When these regions are demethylated, the expression of astrocytic genes is promoted by the activated STAT3, thereby causing astrogenesis [50]. In support of this model, deletion of *Dnmt1* induces early differentiation of NSCs into astrocytes in the neurogenic phase [51]. How does DNA demethylation of astrocyte-specific genes occur? Notch ligands such as delta-like 1 (Dll1) and jagged1 (Jag1) are expressed in NSC-produced neuroblasts or immature neurons, and they activate Notch signaling, which triggers the expression of nuclear factor IA (Nfia) in adjacent NSCs. Nfia binds to the astrocyte-specific gene promoters and detaches Dnmt1 from the promoters, resulting in the demethylation of the promoters [52]. These facts indicate that Notch signaling in NSCs is more strongly activated as neurogenesis progresses, leading to Nfia expression during later embryonic periods. Indeed, Nfia expression is highest in NSCs at around E14, which, surprisingly, correlates with a period of active neurogenesis, inducing DNA demethylation, and setting the epigenetic landscape of NSCs to subsequently produce the cell type astrocytes [53] (Figure 2).

The Ten-eleven translocation (Tet) family is a group of recently identified demethylases that can modify DNA by hydroxylating 5-methylcytosine (5-mC) to 5-hydroxymethylcytosine (5-hmC), an interim phase of the DNA demethylation process [54]. TET proteins further oxidize 5hmC to 5-formyl cytosine (5fC) and 5-carboxyl cytosine (5caC). These oxidized cytosines are successively removed by thymine-DNA glycosylase (TDG), and subsequently replaced by unmodified cytosine through the base excision repair pathway [54,55,56,57]. During neurogenesis in the mouse embryonic brain, the 5hmC level increased in the gene bodies of neuron-specific genes as differentiation progressed, and it was inversely correlated with H3K27 methylation levels [58]. Intriguingly, enrichment of 5hmC was not followed by substantial DNA demethylation, suggesting that 5hmC also served as a stable epigenetic mark. Moreover, the knockdown of Ezh2 promotes the neuronal differentiation of NSCs during development, while such differentiation is enhanced by the overexpression of Tet2 and Tet3, raising the possibility that a gain of 5hmC and reduction of H3K27me3 play important roles in brain development. Similarly to the increase observed in 5hmC levels, 5caC accumulates transiently in the genome, particularly during NSC differentiation [59]. Moreover, this accumulation of 5caC becomes more noticeable when NSCs are prompted to differentiate into glial cells. The knockdown of TDG led to elevated levels of 5caC and 5fC in glia-related promoters during glial differentiation. Regardless of the mechanism involved, these results uphold the idea that DNA demethylation determines the timing of gliogenesis during brain development. Recently, based on a genome-wide CRISPR-Cas9 screen, QSER1 was identified as a DNA methylation regulator, maintaining low methylation at DNA methylation valleys (DMVs), which overlap with developmental genes and broad H3K27me3 and Ezh2 peaks [60]. QSER1 cooperates with TET1 to safeguard transcriptional and developmental programs from DNMT3-mediated de novo methylation at important genomic loci, especially in DMVs and bivalent promoters, where hypermethylation has been linked to developmental disorders and cancers. These findings have provided mechanistic insights into the region-specific regulation of TET pathways in complex brain development.

### 2.3. RNA Modification during Cortical Development

N6-methyladenosine (m6A) is the most abundant internal mRNA methylation in eukaryotes, which has recently been studied as a key regulator of cell fate determination in various neurodevelopmental contexts. Approximately 25% of mammalian mRNAs contain m6A, with an average of 1–3 modifications per transcript [61,62]. For mRNA and other RNA Polymerase II-synthesized transcripts, including most small RNAs and microRNAs (miRNAs), the METTL3–METTL14–WTAP methyltransferase complex is mainly responsible for m6A deposition in newly synthesized transcripts in the cell nucleus [63]. m6A was found to be enriched in conserved locations in the 3′ untranslated regions (UTRs) and near stop codons of transcripts that regulate differentiation and development, whereas it is relatively reduced in housekeeping transcripts [61,62].

Epitranscriptomic regulation via m6A RNA modification is known to affect multiple steps during cortical development. m6A depletion by Mettl14 knockout or Mettl3 knockdown in embryonic mouse brains prolongs the cell cycle progression of NSCs [64]. Since m6A-tagged mRNAs are more easily degraded, deficiency of m6A modification leads to the accumulation of mRNAs related to temporal and cell-type-specific transcription factors, resulting in delayed transitions in developmental competency, including the deep-upper layer neuron transition and the neurogenic–gliogenic transition. Interestingly, m6A partially regulates histone modification (H3K27ac, H3K27me3, and H3K4me3) by destabilizing transcripts that encode histone-modifying enzymes, suggesting potential interactions between epigenetic and epitranscriptomic regulation [65]. m6A reader YTH domain family 2 (YTHDF2) is an m6A-binding protein that affects the localization and stability of targeted mRNA. YTHDF2 promotes the decay of m6A-tagged mRNA by recruiting the CCR4-NOT deadenylase complex through direct interaction with the CNOT1 subunit [66]. Loss of YTHDF2 in the developing mouse brain causes accumulation of neural development-related mRNA and defects in the self-renewal and differentiation capability of NSCs [67]. The RNA-binding protein fragile X mental retardation protein (FMRP) is encoded by the fragile X mental retardation gene (*FMR1*), and mutations in this gene result in Fragile X syndrome, a major genetic cause of intellectual disability. A recent study showed that FMRP is another m6A reader and that it promotes the nuclear export of m6A-tagged mRNA targets during cortical neurogenesis [68]. Interestingly, genetic knockout of *Fmr1* leads to delayed neural progenitor cell cycle progression, like the defect in *Mettl14* knockout mice. These results suggest that selective degradation of m6A-tagged mRNA is essential for proper temporal progression and transcriptional pre-patterning of NSCs during brain development.

## 3. Outline of Adult Neurogenesis and Epigenetic Modifications

Even after the completion of brain development, a limited population of NSCs continues to exist in the adult brain. These NSCs play an important role in brain functions by producing neurons daily. Adult neurogenesis takes place in two specific brain regions (Figure 3 and Figure 4) [9,69,70,71,72,73]. One of these regions that maintains NSCs into adulthood is the SGZ of the adult hippocampal DG (Figure 3). Radial glia-like cells that are relatively quiescent, referred to as type 1 cells, function as NSCs within the SGZ. These type 1 cells produce proliferative NPCs known as type 2a/b cells, which subsequently undergo differentiation into neurons in the neuroblast phase (type 3 cells) (Figure 3). The newborn neurons migrate alongside blood vessels and become part of the existing neural networks in the granule cell layer [73]. These newly formed neurons play a role in various hippocampal functions, including those associated with learning and memory processes dependent on the hippocampus.

Another of these brain regions is the SVZ of the lateral ventricle, where NSCs, known as type B cells, display infrequent proliferation and mostly remain in a quiescent state in the cell cycle [74] (Figure 4). During neurogenesis, they give rise to proliferating NPCs known as type C cells, which then undergo proliferation and differentiate into neuroblasts, referred to as type A cells. These neuroblasts migrate through the rostral migratory stream into the olfactory bulb (OB), where they differentiate into mature granules and periglomerular neurons (Figure 4).

For adult neurogenesis to take place, NSCs must undergo various processes, including the maintenance and proliferation of NS/PCs, as well as neuronal commitment, migration, and maturation [70,74,75]. An increasing body of research has revealed that these processes in adult neurogenesis are regulated by both external and internal cellular factors, such as cytokines released from niche cells, transcription, and epigenetic factors expressed in NSCs [69,73,74,75]. Since NSCs must be sustained from embryonic to adult stages to maintain life-long neurogenesis, the mechanism of sustaining NSCs into adulthood is crucial for neurogenesis in the adult brain. Recent research studies have shown the importance of histone modifications in controlling these processes to support adult neurogenesis [74,75,76]. In this section, we present examples of epigenetic mechanisms underlying the regulation of neurogenesis in the adult brain (Figure 3 and Figure 4).

### 3.1. Histone Modifications for the Control of NS/PC Behavior in the Adult Brain

The PcG and Trithorax (TrxG) complexes are recognized for their pivotal roles in governing adult neurogenesis. The PcG complex orchestrates gene repression via H3K27 methylation, whereas the TrxG complex promotes the expression of target genes by inducing H3K4 trimethylation in promoter-proximal nucleosomes [27,77,78]. B cell-specific Moloney murine leukemia virus integration site 1 (Bmi1), a component of the PRC1 complex, helps to regulate the proliferation rate of NSCs and maintain them into the adult stage. Bmi1 deletion diminishes NSC proliferation, leading to their depletion during development [75]. This effect correlates with the increased expression of p16Ink4a (a cell cycle-dependent kinase inhibitor), and its removal partially restores the phenotype of Bmi1 deficient mice. In relation to the TrxG complex, a histone methyltransferase, mixed-lineage leukemia 1 (Mll1) is indispensable for the neuronal differentiation of NSCs in the SVZ [74,77]. Although Mll1 deletion in NSCs does not affect NSC proliferation, survival, or glial differentiation, it severely impairs neuronal differentiation. Mll1 mediates H3K4me3 and is recruited to gene promoters to induce gene activation. Notably, Mll1 binds directly to the *distal-less homeobox 2* (*Dlx2*) gene locus, a critical transcription factor for neurogenesis from NSCs in the SVZ. When Mll1 is lacking, the *Dlx2* promoter adopts a bivalent state defined by both repressive (H3K27me3) and active (H3K4me3) histone modifications, hindering proper neuronal differentiation of NSCs. This fact indicates the importance of the transition from this poised state (H3K4me3 and H3K27me3) to the active state (H3K4me3 only) at the *Dlx2* promoter for proper neurogenesis. A potential mechanism for Mll1’s function is to cooperate with H3K27me3 demethylase, Jumonji domain-containing 3 (Jmjd3; also known as Kdm6b), to induce target gene expression. Jmjd3 stimulates the expression of various genes associated with the neuronal differentiation of the adult NSC, including *doublecortin*, *Nkx2.2*, *Dlx2*, and *Dlx5* [79,80]. During neuronal differentiation, Jmjd3 localizes to both the promoter and enhancer of *Dlx2*, reducing H3K27me3 levels and enhancing *Dlx2* expression. Interestingly, Jmjd3 recruitment is hindered in the absence of Mll1, indicating that Mll1 is required for recruiting Jmjd3 to its target genomic region. These findings underscore the importance of the interplay between histone methylation enzymes in adult neurogenesis.

Various physiological and pathological circumstances affects adult neurogenesis. One such pathological condition is the induction of seizures by kainic acid, which results in abnormal neurogenesis in the adult hippocampus [81,82,83,84]. The HDAC inhibitor VPA, widely utilized as an antiepileptic drug in human clinical practice, suppresses the abnormal proliferation of NS/PCs induced by kainic acid in the adult DG, thus ameliorating cognitive deficits in hippocampus-dependent learning [83,84]. Moreover, VPA promotes the neuronal differentiation of NSCs in the adult hippocampus by increasing the expression of the proneural gene NeuroD while inhibiting oligodendrocyte and astrocyte differentiation [44].

Within the HDAC family, HDAC2 is essential for neuronal maturation in both the DG and SVZ of the adult hippocampus; the specific loss of HDAC2 in adult NSCs inhibits neuronal differentiation and maturation and leads to cell death in both the SVZ and SGZ [85]. This is due to the persistent expression of stem cell genes in the neurogenic process. SRY-box transcription factor 2 (Sox2), an essential transcription factor for NSC proliferation and the maintenance of NSC stemness, is normally not expressed in neuroblasts committed to neuronal differentiation. However, in HDAC2-deficient mice, Sox2 expression persists in neuroblasts, indicating that HDAC2 is important in arresting Sox2 expression during the differentiation of NS/PCs into neurons [85]. Sox2 also restricts the activity of the PRC2 complex, suppressing excessive acquisition of H3K27me3 in the regulatory regions of proneural and neurogenic genes such as *Neurog2*, *NeuroD1*, and *brain-derived neurotrophic factor* (*BDNF*) [86]. These genes exhibit bivalency at their promoters, poised to activate upon NSCs responding to neurogenic cues and initiating neuronal differentiation. Sox2 binds to these gene promoters and prevents the access of Ezh2, a component of PRC2. Sox2 downregulation results in the accumulation of Ezh2 and elevated H3K27me3 levels at the promoters of the poised proneural gene and neurogenic gene. Furthermore, the Wnt signal, a key inducer of neurogenesis that directs neurogenesis at both adult and embryonic stages, activates the expression of poised proneural genes and neurogenic genes at specific loci. However, NSCs lacking Sox2 fail to express these genes even when stimulated with Wnt. Specific depletion of Sox2 in adult hippocampal NSCs inhibits the expression of proneural/neurogenic genes leading to increased cell death, reduced neurogenesis, and the generation of new neurons exhibiting abnormal functions [86].

### 3.2. DNA Methylation and Demethylation in Adult Neurogenesis

The significance of DNA methylation in adult neurogenesis remains uncertain when compared to other epigenetic regulatory mechanisms such as histone modifications and non-coding RNAs. Dnmt1 and Dnmt3a (3b is not expressed) are expressed in the adult brain. Dnmt1 shows higher expression levels in proliferating NS/PCs than in quiescent NSCs or post-mitotic neurons and astrocytes in the adult hippocampus [87]. The loss of Dnmt1 in adult NSCs does not affect their proliferation and differentiation but reduces the viability of newly formed neurons in adult hippocampal DGs [87]. This suggests that DNA methylation maintained by Dnmt1 at the NSC stage is only responsible for the survival of newborn neurons after NSCs differentiate into neurons in the adult hippocampus. In contrast, Dnmt3a is known to be associated with the neuronal differentiation of NSCs in the adult brain [88]. The deletion of Dnmt3a results in a decline in the neuronal differentiation of NSCs in the SVZ, leading to a decrease in the migration of neuroblasts into the OB. Dnmt3a acts on non-promoter regions of neurogenic genes, stimulating their expression. Methylation by DNA and the presence of Dnmt3a in these non-promoter regions exhibit a negative correlation with the increase in H3K27me3 and the accumulation of PRC2 components Ezh2 and Suz12. This indicates that Dnmt3a-mediated methylation promotes neuronal differentiation by inhibiting PRC2-mediated gene repression. A recent study revealed that deleting both Dnmt3a and Dnmt3b in adult NSCs specifically inhibits dendritic outgrowth and synapse formation in newly formed neurons, thus impeding their functional maturation. Thus, inhibiting de novo DNA methylation modulates the activation pattern of the hippocampal circuit and causes specific impairments of hippocampal-dependent learning and memory [89].

Apart from DNA methyltransferases, Tet1 is a key component that promotes active DNA demethylation via 5mC oxidation and also functions as a pivotal regulator of adult hippocampal neurogenesis [90]. Transcriptome analysis of Tet1-deficient adult neural stem cells (aNSCs) showed that Tet1 demethylates the promoter regions of genes involved in aNSC proliferation and neurogenesis, such as Galamin, Ng2, and Neirglobin, thereby promoting the expression of these genes. Furthermore, spatial learning and memory were impaired in mice lacking Tet1, demonstrating the importance of Tet1-mediated DNA demethylation in cognitive function. Tet2 also contributes to adult neurogenesis. Recently, it was reported that Tet2 expression and 5hmC levels in the hippocampal dentate gyrus decreased with aging. It was also shown that inhibition of Tet2 expression in hippocampal NSCs of adult mice mimicking the aged state resulted in reduced neurogenesis and impaired learning and memory. The overexpression of Tet2 in hippocampal neural niches in aged mice increased 5hmc, offsetting the rapid decline in neurogenesis associated with aging and enhancing learning and memory. Taken together, these results suggest that Tet2 expression is involved in the age-related decline in adult neurogenesis and cognitive and learning functional impairments [91].

Active DNA demethylation occurs in cells comprising the NS/PC niche as well as NS/PC and contributes to the regulation of neurogenic cue expression. Gadd45b, a member of the Gadd45 gene family, is involved in the regulation of neurogenic cue expression by electroconvulsive therapy and voluntary running. The expression of Gadd45b in mature hippocampal neurons relies on depolarization-induced calcium influx and the activity of calmodulin kinase [92,93]. Gadd45b is involved in DNA demethylation via DNA excision repair and promotes active DNA demethylation. In the mature hippocampus of mice, Gadd45b promotes the removal of methyl groups from DNA, which is initiated by neuronal activity in the regulatory region IX of BDNF and the brain-specific promoter B of fibroblast growth factor 1 (FGF1). These genes play vital roles in the growth, survival, and development of neurons in adults. Thus, the process of DNA demethylation facilitated by Gadd45 and the secretion of proteins such as BDNF and FGF1 contribute to the regulation of the environment conducive to neurogenesis in adulthood, acting as important controllers of balance in adult brain cell production.

DNA methylation usually results in gene repression by recruiting methyl-CpG-binding protein 1 (MBD1) and methyl-CpG-binding protein 2 (MeCP2), members of the methylated DNA binding protein family. These two proteins also play a role in regulating the expression of neurogenic cues. MBD1-deficient mice show decreased neurogenesis in the adult hippocampus and impaired spatial learning [94]. Mechanistically, MBD1 represses the transcription of miR-184 by binding to the methylated miR-184 promoter region. miR-184 regulates the proliferation and differentiation of aNSCs by suppressing the expression of Numblike (Numbl), which is known to inhibit Notch signaling. This MBD1-mediated miR-184/Numbl cascade regulates the balance between the proliferation and differentiation of aNSCs [95]. RNA sequencing (RNA-seq) using MBD1-deficient aNSCs also revealed an increased expression of astrocyte-specific genes [96]. These results suggest that MBD1 maintains the transcriptional integrity of aNSCs and supports epigenetic mechanisms that fine-tune fate decisions. MeCP2, the gene responsible for Rett syndrome, exhibits significant expression in postmitotic neurons and serves as a key controller of neuronal gene expression within the central nervous system [97,98,99]. MeCP2 knockout mice display hindered neuronal maturation in newly formed neurons within the adult hippocampal DG [100]. Among MeCP2’s well-recognized targets is BDNF, which governs various facets of neurogenesis, including the proliferation and differentiation of NSCs, as well as the growth and viability of emerging neurons [101,102]. MeCP2 binds to the hypermethylated BDNF promoter and suppresses its expression. This interaction between MeCP2 and the BDNF promoter is disrupted by the induction of DNA demethylation in response to neuronal activity, leading to increased BDNF transcription. Additionally, neuronal activity-induced calcium influx causes posttranscriptional modification of MeCP2, such as phosphorylation at Ser421, which reduces the affinity of MeCP2 for the BDNF promoter, resulting in the derepression of the BDNF gene [103,104]. The phosphorylation of MeCP2 at Ser421 is triggered by neuronal stimulation and plays significant roles in synaptic development and behavior [105]. Factors associated with DNA methylation and demethylation and their effects on adult neurogenesis are summarized in Table 2.

## 4. Epigenetic Dysregulation in Brain Disorders

Recent evidence has suggested that epigenetic dysregulation contributes to the pathogenesis of a variety of neurological disorders [106].

Parkinson’s disease (PD) is a common and progressive neurodegenerative disorder characterized by classical motor dysfunction and associated with alpha (α)-synuclein abnormalities and loss of dopaminergic neurons in the substantia nigra [107,108,109]. Most PD patients exhibit high levels of α-synuclein accumulation in the brain, and it has been reported that this accumulation increases cell death and impairs the dendritic development of newborn neurons in the adult mouse hippocampus [110]. Overexpression of SNCA (which encodes α-synuclein) is associated with the hypomethylation of CpG islands in intron 1 of SNCA [111,112]. In the brains of deceased individuals with PD and in the brains of mouse PD models with SNCA transgenes, DNMT1 undergoes a systematic transition from the nucleus to the cytoplasm [113]. The reduction of nuclear DNMT1 is linked to the hypomethylation of numerous genes associated with PD, including SNCA. The relocation of DNMT1 is a consequence of its sequestration by α-synuclein, and partial restoration of nuclear Dnmt1 levels in transgenic mouse brains is achieved through Dnmt1 overexpression [114,115]. These findings suggest that epigenetic dysregulation of PD-related genes may contribute to PD symptoms through impaired adult neurogenesis.

Epigenetic dysregulation of adult neurogenesis is also involved in Alzheimer’s disease (AD) [116]. Genetic studies have identified mutations in three genes: amyloid precursor protein (APP), presenilin 1 (PSEN1), and PSEN2, mainly causing early-onset AD, while polymorphisms in apolipoprotein E (APOE) are linked to late-onset AD [117,118]. Accumulating evidence now suggests that the epigenetic dysregulation of APP, PSEN1, PSEN2, APOE, and/or MAPT (which encodes tau) potentially contributes to the pathogenesis of AD [119]. For instance, there have been reports of a widespread reduction in DNA methylation and 5hmC levels in the hippocampus of AD patients [120]. A recent comprehensive genome-wide analysis of DNA methylation in AD brains identified altered DNA methylation states in 71 specific CpG dinucleotides, accompanied by the dysregulated expression of associated genes [121]. It is well established that PSEN1 plays a crucial role in neurodevelopment and differentiation; the absence of Psen1 leads to premature differentiation of neural progenitor cells [122]. Moreover, the loss of Psen1 results in learning and memory deficits in mice, likely due to impaired neurogenesis in the adult hippocampus [123]. During development, the expression of Psen1 in the cerebral cortex is controlled by promoter DNA methylation in coordination with H3K9 acetylation [124]. In Apoe-knockout mice, hyperactive BMP signaling promotes glial differentiation during adult neurogenesis [125]. Furthermore, hypermethylated CpG islands at the 3′ end of APOE serve dual regulatory roles as either enhancers or silencers, influencing the transcription of multiple genes, including APOE, Translocase Of Outer Mitochondrial Membrane 40, Neural Precursor Cell Expressed, and Developmentally Down-Regulated 9 (TOMM40, and NEDD9) [126].

Huntington’s disease (HD), a progressive brain disorder caused by 41 or more CAG trinucleotide repeat expansions in huntingtin (HTT) [127], is characterized by mutant HTT protein aggregates in and around the nucleus of neurons [128]. Transgenic mice expressing mutant HTT exhibit reduced NPC proliferation in the adult dentate gyrus, resulting in a decrease in the number of newly generated neurons [129,130]. Cumulative evidence suggests that epigenetic modulation of HTT plays a significant role in neurogenesis in HD [131,132]. Epigenetic changes could thus potentially influence the expression and elongation of HTT directly, or mutant HTT may interact with various epigenetic modulators, leading to the modifications of epigenetic states. For instance, methylation of CAG repeats prevents the generation of repeat expansions in vitro. Additionally, treatment of cells with DNMT inhibitors causes extensive demethylation and promotes the generation of repeat expansions during replication [133]. Furthermore, a genome-wide DNA methylation analysis reveals that the expression of mutant HTT leads to significant changes in DNA methylation and the transcriptome, affecting many genes essential for neurogenesis, including downregulating Pax6 and Nestin in HD transgenic mice [134].

Amyotrophic lateral sclerosis (ALS) and frontotemporal dementia (FTD) are complex disorders characterized by genetic and pathological diversity involving a multitude of genetic elements [135,136]. The expansion of the GGGGCC (G4C2) repeat in the chromosome 9 open reading frame 72 (C9orf72) gene represents the most prevalent genetic anomaly associated with ALS and FTD. While ALS primarily manifests as the degeneration of motor neurons [137], FTD involves the loss of neurons in the frontal and anterior temporal lobes of the brain [138]. The C9orf72 gene comprises 11 exons, with ≤11 hexanucleotide G4C2 repeats typically found in the majority of neurologically healthy individuals. However, in ALS/FTD cases, pathogenic expansion of these repeats within intron 1 disrupts normal gene function, leading to C9orf72 haploinsufficiency through mechanisms involving both loss of function and gain of toxic properties. Additionally, hypermethylation of G4C2 repeats, observed in approximately 97% of ALS/FTD patients with more than 50 repeats, further contributes to the dysfunction of the C9orf72 protein by inhibiting the transcriptional activity of its gene [139].

Epilepsy is a chronic neurological disorder in which highly synchronized and high-frequency activity of neurons leads to an epileptic seizure [140]. This activity causes abnormal electrical discharges in the brain, resulting in a variety of symptoms including loss of consciousness and generalized convulsions [141]. Epilepsy can be caused by a variety of factors, including genetic factors, brain damage, metabolic abnormalities, and brain tumors, and epigenetic changes may be involved in the mechanism [142,143,144]. AMPA receptors are hetero-oligomers composed of four molecular subunits termed GluR1–4. Previous studies have reported that GluR2 expression in neurons is reduced in various neurological disorders, including prolonged seizures, which is thought to result in pathologically increased toxic Ca^2+^ entry via AMPA receptors [145]. This process is epigenetically regulated through a decrease in histone H4 acetylation in GluR2 [145]. Similarly, acetylation regulates several processes related to epilepsy progression, including increased H4 acetylation at the BDNF P2 promoter after status epilepticus superimposition [145] and activation of c-fos and c-jun expression during epileptogenesis in the hippocampus [146]. VPA has commonly been used as a treatment for epilepsy. VPA enhances the inhibitory activity of γ-aminobutyric acid (GABA) in both pre- and postsynaptic mechanisms and promotes synaptic GABA availability, resulting in the attenuation of neuronal activity [147,148]. In addition, VPA has HDAC inhibitory activity and suppresses abnormal neurogenesis that may induce cognitive dysfunction in epilepsy [82]. Therefore, it is feasible that VPA protects animals from seizure-induced cognitive dysfunction in hippocampus-dependent learning tasks by inhibiting ectopic neurogenesis associated with epileptogenesis through its activity as an HDAC inhibitor [82,149].

## 5. Conclusions and Perspective

Understanding the complex interplay of histone, DNA, and RNA modifications is essential for unraveling the regulatory mechanisms governing cortical development. These epigenetic processes contribute to the precise timing and coordination of neurogenesis and astrogenesis, laying the foundation for proper brain development. Understanding how these epigenetic marks regulate gene expression programs during critical periods of neurodevelopment may provide important insights into the etiology of neurodevelopmental disorders and help to develop targeted interventions. Furthermore, technological advances such as single-cell sequencing and spatial transcriptomics offer unprecedented opportunities to dissect the epigenetic landscape of individual cell types and regions within the developing brain. By characterizing cell-type-specific epigenomic profiles and gene regulatory networks, researchers can gain deeper insights into the molecular mechanisms driving neural differentiation, circuit formation, and synaptic plasticity. Investigating the interplay between epigenetics and environmental factors in shaping brain development represents a fertile area for future research. Studies exploring how prenatal exposure to environmental toxins, maternal stress, nutrition, and early-life experiences influence epigenetic programming in the developing brain could shed light on the mechanisms underlying neurodevelopmental disorders and identify potential avenues for preventive strategies.

In addition to regulating brain development, epigenetic mechanisms also regulate adult neurogenesis. The PcG and TrxG complexes orchestrate gene expression, while key players such as Bmi1, Mll1, Dnmt1, Dnmt3a, Tet1, and Tet2 modulate specific aspects. Dysregulation of these epigenetic processes is implicated in neurological disorders, underscoring their importance in maintaining brain health. Recently, the field of “epigenetic editing” holds promise for modulating gene expression patterns in a precise and reversible manner. Techniques such as CRISPR-based epigenome editing enable targeted manipulation of epigenetic marks, opening up new possibilities for correcting aberrant epigenetic states associated with neurodevelopmental and neurodegenerative disorders. Moreover, translating findings from basic research into clinical applications is a crucial next step in harnessing the potential of epigenetics for brain health. Developing epigenetic-based biomarkers for early detection, diagnosis, and prognosis of neurological disorders, as well as designing personalized epigenetic therapies tailored to individual patients, could revolutionize the treatment landscape and improve outcomes for those affected by neurodevelopmental conditions. In conclusion, the future of research in epigenetics and brain development control has great potential. By continuing to unravel the complexities of epigenetic regulation in the developing brain, researchers can pave the way for innovative therapeutic strategies and personalized interventions that will hopefully improve the lives of patients with neurological disorders.

## Figures and Tables

**Table 1:** Histone modification-related genes controlling NSC properties during cortical development.

Genes	Type of Histone Modification	Function	References
Ezh2	Histone H3K27 methyltransferase	NSC differentiation	[28,29,30,31]
Setdb1	Histone H3K9 methyltransferase	NSC differentiation	[32,33]
LSD1	Histone H3K4 and H3K9 demethyltransferase	NSC proliferation	[34,35]
PRDM16	Histone H3K4 and H3K9 methyltransferase	NSC proliferation	[40,41]
HDAC2, 5	Histone deacetylase	NSC proliferation, NSC differentiation	[37,42]

**Table 2:** DNA modification-related genes during adult neurogenesis.

Genes	Type of DNA Modification	Function for Adult Neurogenesis	References
DNMT1	Maintenance DNA metyltransferase	Immature neuron survival	[87]
DNMT3A	De novo DNA methyltransferase	Neuron differentiation, Neuronal maturation	[88]
TET1	5-methylcytosine hydroxylase	aNSCs proliferation, Neuronal differentiation	[90]
TET2	5-methylcytosine hydroxylase	aNSCs proliferation, Neuronal differentiation	[91]
MBD1	Methyl-CpG binding protein	qNSCs maintenance, Neuronal differentiation	[94]

qNSCs: quiescent neural stem cells, aNSCs: active neural stem cells.

## Data Availability

Not applicable.
